# Interleukin 17A in Patients With Stable Coronary Artery Disease: Are There Differences According to Gender?

**DOI:** 10.14740/cr363w

**Published:** 2014-12-04

**Authors:** Dinaldo Oliveira, Elayne Heide, Carlos Brandt, Moacyr Rego, Maira Pitta, Ivan Pitta

**Affiliations:** a Hospital of Clinics, Federal University of Pernambuco, Av. Prof. Moares Rego, 12 35, CEP 50670-901, Recife, Pernambuco, Brazil; b Center of Biological Science, Immunomodulation Laboratory and New Therapeutic Approaches, Federal University of Pernambuco, Av. Prof. Moares Rego, 12 35, CEP 50670-901, Recife, Pernambuco, Brazil

**Keywords:** Coronary artery disease, Interleukin 17A, Inflammation, Immunity

## Abstract

**Background:**

Coronary artery disease (CAD) is a current major public health concern. Immunity and inflammation are involved in all phases of CAD and there is a dynamic balance between cells and molecules. Interleukin 17A (IL17A) concentrations are higher in male patients with acute myocardial infarction than in women. In this study, we evaluated if the IL17A concentrations in male CAD patients (MPs) differed from those in female patients (FPs) and male controls (MCs). Moreover, FPs were compared with female controls (FCs).

**Methods:**

This was a cross-sectional, prospective, and analytical study conducted between March 2012 and August 2013 that enrolled 40 patients (24 men and 16 women) with stable CAD and 20 healthy volunteers (12 men and 8 women) were selected as controls and were matched with the patients (1:2) for sex and age (± 3 years). Comparative analyses of IL17A concentrations in serum and cell culture with and without stimulation were performed between MPs and MCs, MPs and FPs, and FPs and FCs. The lower detection limit was 3.91 pg/mL.

**Results:**

The comparison of the IL17A concentrations showed: after 48 hours of cell culture with stimulus: MP = 451.67 (99.02 - 892.58) vs. MC = 135 (3.91 - 285), P = 0.04; after 48 hours of cell culture with stimulus: MP = 451.67 (99.02 - 892.58) vs. FP = 131.21 (3.91 - 231.97), P = 0.02; after 48 hours of cell culture with stimulus: FP = 131.21 (3.91 - 231.97) vs. FC = 173.78 (3.91 - 642), P = 0.24.

**Conclusion:**

This study revealed higher IL17A concentrations in the stimulated cells isolated from the MPs than in those isolated from FPs and MCs. These findings support the hypothesis that when exposed to certain stimuli, cells isolated from MPs with chronic CAD may produce higher IL17A concentrations than those from FPs and MCs.

## Introduction

Coronary artery disease (CAD) is a current major public health concern. Its etiology and pathophysiological mechanism are complex and, as such, have been the focus of several studies over the past 50 years [1, 2].

Immunity and inflammation are involved in all phases of CAD and there is a dynamic balance between cells and molecules. Although knowledge in this area has increased in recent years, further research is required [3, 4].

The incidence and prognosis of CAD usually differs between men and women. Anatomical and pathophysiological aspects, as well as response to the treatment of coronary atherosclerosis, differed when patients with CAD were analyzed according to sex [1, 5, 6].

Regarding the immune response of CAD patients, while the activation of the Th1 pathway has been established, several aspects regarding the involvement of Th2 and Th17 remain controversial [7].

Although CAD is a single disease, the morphofunctional aspects of atherosclerotic plaques and clinical manifestations greatly vary, thus making it possible to correlate it with several aspects of patients’ immune response [8].

In this study, we evaluated whether the interleukin 17A (IL17A) concentrations in male CAD patients (MPs) differed from those in female patients (FPs) and male controls (MCs). Moreover, FPs were compared with female controls (FCs).

## Materials and Methods

This was a cross-sectional, prospective, and analytical study conducted between March 2012 and August 2013. This study was performed in accordance with the ethical principles of clinical research and was approved by the clinical research ethics committee of the institution where it was performed (in accordance with the ethical standards laid down in the 1964 Declaration of Helsinki and its later amendments).

This study enrolled 40 stable CAD patients (24 men and 16 women) with Canadian Cardiovascular Society functional class III or IV: patients with ischemia on myocardial scintigraphy (under optimized medical treatment) but who had not undergone myocardial revascularization; who had at least one coronary stenosis ≥ 50% on coronary angiography performed on the recruitment day; patients with no severe liver or lung disease, cancer, or rheumatic or immune disorders; patients with no acute disease in the previous 3 months; and patients who agreed to participate in the study by signing the consent form.

Twenty healthy volunteers (12 men and 8 women) were selected as controls and were matched with the patients (1:2) for sex and age (± 3 years).

Stenoses were categorized as moderate (50-69% obstruction) and severe (≥ 70% obstruction). The patients were categorized according to the number of arteries with stenosis: single-artery disease (one artery with stenosis ≥ 50%), two-artery disease (two arteries with stenosis), or multi-artery disease (≥ 3 arteries with stenosis).

A 10-mL sample of peripheral blood was collected from the patients and controls immediately after recruitment and sent to the laboratory for enzyme-linked immunosorbent assay (ELISA) and cell culture.

Mononuclear cells were extracted from the heparinized peripheral blood samples from the patients and controls. These cells were then isolated using the standard Ficoll-Hypaque density gradient centrifugation method. The cells were placed in a Neubauer chamber, and cell viability was determined using the trypan blue exclusion method. Cells were used when the viability was > 98%. Comparisons were made after 48 h of culture with and without stimulation using CD3 and CD28. The lower detection limit was 3.91 pg/mL.

In this study, comparative analyses of IL17A concentrations in serum and after 48 h cell culture with and without stimulation were performed between MPs and MCs, MPs and FPs, and FPs and FCs.

The categorical variables were shown as percentages, and the numerical variables as median (maximum and minimum values) or media (standard deviation).

Descriptive statistical analysis was performed to describe the clinical variables and complementary tests in the study. The Kolmogorov-Smirnov normality test was used for quantitative variables, and the Mann-Whitney nonparametric test was used to compare the groups (analytical statistics) when the distribution was not normal. Ages were compared using the Student’s *t*-test. Categorical variables were compared using the Chi-square test. A P value ≤ 0.05 was statistically significant.

## Results

The study enrolled 40 patients (24 men and 16 women) and no difference in mean age was observed between sexes (61.4 ± 9.4 vs. 66.5 ± 7.1 years; P = 0.07). The controls included 12 men and 8 women. Similarly, no difference was observed in their mean ages (57.5 ± 12.5 vs. 58.6 ± 7.4 years, P = 0.8).

Table 1 shows the comparison of the main clinical characteristics between men and women (patients).

**Table 1:** Clinical Characteristics of the Patients

Comorbidities	Men (24 patients)	Women (16 patients)	P
Hypertension, n (%)	14 (59%)	8 (50%)	0.8
Dyslipidemia, n (%)	11 (45.8%)	8 (50%)	0.9
Diabetes mellitus, n (%)	6 (25%)	9 (56.2%)	0.09
Smoking, n (%)	4 (16.6%)	3 (18.7%)	0.7
Peripheral artery disease, n (%)	2 (8.3%)	3 (18.7%)	0.6
Stroke, n (%)	1 (4.1%)	1 (6.2%)	0.6
Chronic kidney disease, n (%)	1 (4.1%)	1 (6.2%)	0.6

In the male group, stenosis ≥ 50% occurred in left descendent anterior (LAD) in 21 patients (87.5%), in left circumflex (LCX) in 11 (54.8%), and in right coronary artery (RCA) in 16 (66%). No stenosis was observed in left main (LM). In the female group, stenosis ≥ 50% occurred in LAD in 10 patients (41.6%), in LCX in eight (33.3%), and in RCA in eight (33.3%). There was not stenosis in LM.

In the comparison between MPs and MCs, there were higher IL17A concentrations in stimulated cells isolated from the patient group (Table 2). However, no significant differences in the IL17A concentrations were observed in the comparative analysis between FPs and FCs (Table 3).

**Table 2:** Interleukin 17A in Male Patients and Male Controls

		Men		P
		Patients	Controls	
		Median (Min - Max)	Median (Min - Max)	
IL17A	Serum	3.91 (3.91 - 3.91)	3.91 (3.91- 9.28)	0.31
	CCWS	3.91 (3.91 - 3.91)	3.91 (3.91 - 3.91)	0.45
	CCS	451.67 (99.02 - 892.58)	135 (3.91 - 285)	0.04

IL17A: interleukin 17A; CCWS: after 48 h of cell culture without stimulation; CCS: after 48 h of cell culture with stimulation.

**Table 3:** Interleukin 17A in Female Patients and Female Controls

		Women		P
		Patients	Controls	
		Median (Min - Max)	Median (Min - Max)	
IL17A	Serum	3.91 (3.91 - 3.91)	3.91 (3.91 - 625)	0.90
	CCWS	3.91 (3.91 - 3.91)	3.91 (3.91 - 3.91)	0.20
	CCS	131.21 (3.91 - 231.97)	173.78 (3.91 - 642)	0.24

IL17A: interleukin 17A; CCWS: after 48 h of cell culture without stimulation; CCS: after 48 h of cell culture with stimulation.

In the comparison between the MPs and FPs, there were higher IL17A concentrations in the stimulated cells isolated from the MPs (Table 4).

**Table 4:** Interleukin 17A in Male Patients and Female Patients

		Patients		P
		Men	Women	
		Median (Min - Max)	Median (Min - Max)	
IL17A	Serum	3.91 (3.91- 3.91)	3.91 (3.91 - 3.91)	0.17
	CCWS	3.91 (3.91 - 3.91)	3.91 (3.91 - 3.91)	0.47
	CCS	451.67 (99.02 - 892.58)	131.21 (3.91 - 231.97)	0.02

IL17A: interleukin 17A; CCWS: after 48 h of cell culture without stimulation; CCS: after 48 h of cell culture with stimulation.

## Discussion

This study revealed higher IL17A concentrations in the stimulated cells isolated from the MPs than in those isolated from FP and MC.

Hashmi et al observed higher IL17 concentrations in patients with acute coronary syndromes than in patients with chronic CAD [9].

Zhang et al showed higher IL17A concentrations in male patients with acute myocardial infarction (AMI) than in women [10].

Our study also revealed higher IL17A concentrations in men than in women; however, it included patients with chronic and stable coronary syndrome.

Several investigations revealed that IL17A concentration did not increase in patients with chronic CAD compared to those in controls [9, 11]. In our study, patients were evaluated according to sex and higher concentrations of the IL17A were observed in MPs than in the MCs and FPs.

There are data revealing that the prevalence of CAD differed according to the sex of the patients. The size of coronary arteries, prevalence of CAD, prognosis, mortality rates, and incidence of complications after CABG or PCI may vary according to the sex of the patient [12, 13].

The anatomical, physiological, and clinical characteristics of CAD differed between male and female patients. The prevalence of AMI is higher among MPs than among FPs. Although several causes to explain this difference have been postulated, the phenomenon is still not completely understood [1, 6, 14].

IL17A concentration has been studied for several years, and its involvement in atherosclerosis has been investigated. The following effects of this interleukin are related to CAD: attraction and activation of neutrophils, synergistic action with the tumor necrosis factor alpha, release of IL6 and IL8, induction of pro-inflammatory changes in different cell types, injury and apoptosis of endothelial cells, influence on the polarization of type 1/type 2 macrophages, combined action with interferon gamma for the production of inflammatory interleukins by smooth muscle cells and synergistic effect with IL12 [15-18].

In animal models, IL17A deficiency was observed to reduce atherosclerosis, decrease the number of macrophages in the atheroma, and inhibit release of monocyte chemoattractant protein 1, IL1b, IL12, and interferon gamma in the plaque. However, administration of this interleukin was also reported to inhibit the formation of atheroma, and its deficiency did not affect atherosclerosis [19-22].

Therefore, IL17A was considered by many investigators as pro-atherosclerotic and, as such, is associated with a possible acute coronary syndrome. Conversely, while other authors reported anti-atherosclerotic effects, many believe that the type of diet administered to the animals partly influences the results, suggesting an anti-atherosclerotic effect [23].

The IL17 may be involved in the disruption of vulnerable plaques triggered by short-term stimulation with lipopolysaccharide, phenylephrine injection and cold in ApoE2/2 mice [24].

In this study, when the cells isolated from the patients with chronic CAD were kept in contact with positive stimulating compounds, higher IL17A concentrations were observed in the MPs than in the MCs and FPs.

These findings support the hypothesis that when exposed to certain stimuli, cells isolated from male patients with chronic CAD may produce higher IL17A concentrations than those from FPs. Moreover, as the concentration of this interleukin was shown to increase in patients with AMI [10], the enhanced IL17A concentration may partly justify the higher incidence of acute coronary syndromes in men.

Therefore, in conclusion these results support the hypothesis that the increased ability of cells from male patients with chronic CAD to produce IL17A after exposure to stimuli may be a factor involved in the development of AMI.

The main limitations of the study are the fact that no evaluation on which cells produced IL17A was performed, absence of dosage of the other interleukins associated with IL17A, possible differences in clinical profile that may not have been found because of the sample size.

## References

[1] Roger VL, Go AS, Lloyd-Jones DM, Benjamin EJ, Berry JD, Borden WB, Bravata DM (2012). Heart disease and stroke statistics--2012 update: a report from the American Heart Association. Circulation.

[2] Libby P, Ridker PM, Hansson GK (2011). Progress and challenges in translating the biology of atherosclerosis. Nature.

[3] Bentzon JF, Otsuka F, Virmani R, Falk E (2014). Mechanisms of plaque formation and rupture. Circ Res.

[4] Libby P, Tabas I, Fredman G, Fisher EA (2014). Inflammation and its resolution as determinants of acute coronary syndromes. Circ Res.

[5] Fihn SD, Gardin JM, Abrams J, Berra K, Blankenship JC, Dallas AP, Douglas PS (2012). 2012 ACCF/AHA/ACP/AATS/PCNA/SCAI/STS guideline for the diagnosis and management of patients with stable ischemic heart disease: a report of the American College of Cardiology Foundation/American Heart Association task force on practice guidelines, and the American College of Physicians, American Association for Thoracic Surgery, Preventive Cardiovascular Nurses Association, Society for Cardiovascular Angiography and Interventions, and Society of Thoracic Surgeons. Circulation.

[6] Otten AM, Maas AH, Ottervanger JP, Kloosterman A, van't Hof AW, Dambrink JH, Gosselink AT (2013). Is the difference in outcome between men and women treated by primary percutaneous coronary intervention age dependent? Gender difference in STEMI stratified on age. Eur Heart J Acute Cardiovasc Care.

[7] Pober JS (2011). Interleukin-17 and atherosclerotic vascular disease. Arterioscler Thromb Vasc Biol.

[8] O'Gara PT, Kushner FG, Ascheim DD, Casey DE, Chung MK, de Lemos JA, Ettinger SM (2013). 2013 ACCF/AHA guideline for the management of ST-elevation myocardial infarction: executive summary: a report of the American College of Cardiology Foundation/American Heart Association Task Force on Practice Guidelines. Circulation.

[9] Hashmi S, Zeng QT (2006). Role of interleukin-17 and interleukin-17-induced cytokines interleukin-6 and interleukin-8 in unstable coronary artery disease. Coron Artery Dis.

[10] Zhang X, Pei F, Zhang M, Yan C, Huang M, Wang T, Han Y (2011). Interleukin-17A gene variants and risk of coronary artery disease: a large angiography-based study. Clin Chim Acta.

[11] Oliveira DC, Heide E, Rego M, Pitta M, Pitta I (2014). Interleukin 17 a expression in chronic coronary artery disease. Exp Clin Cardiol.

[12] Perl L, Bental T, Assali A, Vaknin-Assa H, Lev E, Kornowski R, Porter A (2015). Impact of female sex on long-term acute coronary syndrome outcomes. Coron Artery Dis.

[13] D'Ascenzo F, Gonella A, Quadri G, Longo G, Biondi-Zoccai G, Moretti C, Omede P (2011). Comparison of mortality rates in women versus men presenting with ST-segment elevation myocardial infarction. Am J Cardiol.

[14] Ferrante G, Corrada E, Belli G, Zavalloni D, Scatturin M, Mennuni M, Gasparini GL (2011). Impact of female sex on long-term outcomes in patients with ST-elevation myocardial infarction treated by primary percutaneous coronary intervention. Can J Cardiol.

[15] Zhu F, Wang Q, Guo C, Wang X, Cao X, Shi Y, Gao F (2011). IL-17 induces apoptosis of vascular endothelial cells: a potential mechanism for human acute coronary syndrome. Clin Immunol.

[16] Liu Z, Lu F, Pan H, Zhao Y, Wang S, Sun S, Li J (2012). Correlation of peripheral Th17 cells and Th17-associated cytokines to the severity of carotid artery plaque and its clinical implication. Atherosclerosis.

[17] Csiszar A, Ungvari Z (2004). Synergistic effects of vascular IL-17 and TNFalpha may promote coronary artery disease. Med Hypotheses.

[18] Eid RE, Rao DA, Zhou J, Lo SF, Ranjbaran H, Gallo A, Sokol SI (2009). Interleukin-17 and interferon-gamma are produced concomitantly by human coronary artery-infiltrating T cells and act synergistically on vascular smooth muscle cells. Circulation.

[19] Usui F, Kimura H, Ohshiro T, Tatsumi K, Kawashima A, Nishiyama A, Iwakura Y (2012). Interleukin-17 deficiency reduced vascular inflammation and development of atherosclerosis in Western diet-induced apoE-deficient mice. Biochem Biophys Res Commun.

[20] Yao Z, Fanslow WC, Seldin MF, Rousseau AM, Painter SL, Comeau MR, Cohen JI (1995). Herpesvirus Saimiri encodes a new cytokine, IL-17, which binds to a novel cytokine receptor. Immunity.

[21] Erbel C, Chen L, Bea F, Wangler S, Celik S, Lasitschka F, Wang Y (2009). Inhibition of IL-17A attenuates atherosclerotic lesion development in apoE-deficient mice. J Immunol.

[22] Taleb S, Romain M, Ramkhelawon B, Uyttenhove C, Pasterkamp G, Herbin O, Esposito B (2009). Loss of SOCS3 expression in T cells reveals a regulatory role for interleukin-17 in atherosclerosis. J Exp Med.

[23] Madhur MS, Funt SA, Li L, Vinh A, Chen W, Lob HE, Iwakura Y (2011). Role of interleukin 17 in inflammation, atherosclerosis, and vascular function in apolipoprotein e-deficient mice. Arterioscler Thromb Vasc Biol.

[24] Ma T, Gao Q, Zhu F, Guo C, Wang Q, Gao F, Zhang L (2013). Th17 cells and IL-17 are involved in the disruption of vulnerable plaques triggered by short-term combination stimulation in apolipoprotein E-knockout mice. Cell Mol Immunol.

